# Supplementary material for: Predictors of time to recovery and non-response during outpatient treatment of severe acute malnutrition
Source: PLoS One. 2022 May 31;17(5):e0267538. doi: 10.1371/journal.pone.0267538 (PMC9154090; doi:10.1371/journal.pone.0267538)
Supplement: Attachment — Submitted filename: Response_to_Reviewers_FINAL_new.docx [file pone.0267538.s001.docx]

Dear Editor and Reviewers,

Many thanks for the careful review of our manuscript on the “Predictors of time to recovery and non-response during outpatient treatment of severe acute malnutrition”. We truly appreciated the thoughtful feedback provided and have now worked to improve our manuscript addressing the specific points raised. Please find below an explanation of the improvements made to the manuscript. Our responses are highlighted in ***bold italics***.

Editors comments:

1. Please ensure that your manuscript meets PLOS ONE's style requirements, including those for file naming. The PLOS ONE style templates can be found at

https://journals.plos.org/plosone/s/file?id=wjVg/PLOSOne_formatting_sample_main_body.pdf and

<https://journals.plos.org/plosone/s/file?id=ba62/PLOSOne_formatting_sample_title_authors_affiliations.pdf>

***Response:  Thank you for the orientation. We have now revised the formatting to correspond to the Plos One requirements.***

2. Thank you for stating the following in the Competing Interests section:

'STK was previously employed by Nutriset, a producer of RUTF. HF has received research grants from ARLA Food for Health Centre, and also has research collaboration with Nutriset. Other authors declare no financial relationships
with any organisations that might have an interest in the submitted work in the previous five years, and declare no other relationships or activities that could appear to have influenced the submitted work.'

a. Please confirm that this does not alter your adherence to all PLOS ONE policies on sharing data and materials, by including the following statement: "This does not alter our adherence to  PLOS ONE policies on sharing data and materials.” (as detailed online in our guide for authors [http://journals.plos.org/plosone/s/competing-interests](about:blank)).  If there are restrictions on sharing of data and/or materials, please state these.

Please note that we cannot proceed with consideration of your article until this information has been declared.

***Response:  Thank you for this guidance. We have now revised the conflict of interest statement to include the sentence “The declared competing interests do not alter our adherence to  PLOS ONE policies on sharing data and materials”.***

b. Please include your updated Competing Interests statement in your cover letter; we will change the online submission form on your behalf.

***Response:  Thank you, this has been done.***

Please know it is PLOS ONE policy for corresponding authors to declare, on behalf of all authors, all potential competing interests for the purposes of transparency. PLOS defines a competing interest as anything that interferes with, or could reasonably be perceived as interfering with, the full and objective presentation, peer review, editorial decision-making, or publication of research or non-research articles submitted to one of the journals. Competing interests can be financial or non-financial, professional, or personal. Competing interests can arise in relationship to an organization or another person. Please follow this link to our website for more details on competing interests: [http://journals.plos.org/plosone/s/competing-interests](about:blank)

3. We note that you have included the phrase “data not shown” in your manuscript. Unfortunately, this does not meet our data sharing requirements. PLOS does not permit references to inaccessible data. We require that authors provide all relevant data within the paper, Supporting Information files, or in an acceptable, public repository. Please add a citation to support this phrase or upload the data that corresponds with these findings to a stable repository (such as Figshare or Dryad) and provide and URLs, DOIs, or accession numbers that may be used to access these data. Or, if the data are not a core part of the research being presented in your study, we ask that you remove the phrase that refers to these data.

***Response: Thank you for this clarification. We have thus pursued to suppressing this part of the discussion: ~~More children with both admission criteria reached MUAC ≥125 mm before reaching a WHZ ≥-2 (data not shown). It would thus seem that reaching a WHZ ≥-2 is more challenging for recovering young children and maybe unreachable for some as indicated by the high proportion of non-response to treatment.~~***

4. Please amend your list of authors on the manuscript to ensure that each author is linked to an affiliation. Authors’ affiliations should reflect the institution where the work was done (if authors moved subsequently, you can also list the new affiliation stating “current affiliation:….” as necessary).

***Response: Thank you, this has now been done.***

Reviewers' comments:

5. Review Comments to the Author

Please use the space provided to explain your answers to the questions above. You may also include additional comments for the author, including concerns about dual publication, research ethics, or publication ethics. (Please upload your review as an attachment if it exceeds 20,000 characters)

Reviewer #1: The primary analysis used was the proportional hazard regression models which were fitted to quantify effects of predictors on time to recovery. The Kaplan Meier plots were used to visualize results concerning age and admission categories. Restricted mean time to recovery was estimated for significant categorical predictors. Logistic regression was used to evaluate predictors of non-response to treatment. Both unadjusted and adjusted models for sex and age at admission were fitted. The analysis appears reasonable.

The data is from the MANGO study . The study design and some protocol information is noted on page 4. The authors note on page 7 that excluding the 157 referrals, 644 children contributed to the analysis of time to recovery and all 801 to the analysis of non-response to treatment. The study design lacks statistical justification in the requires sample size from a power perspective. Given the large number of variables considered in the time to event and non response analyses and the large number of p-values presented, some rationale for the sample size is appropriate, despite the descriptive nature of the study. The sample size certainly appears adequate. However, the authors should comment on the statistical aspects of its adequacy.

***Response: Thank you for raising this question. We studied a total of 25 predictors for a total of 433 events resulting in ratio of 17 events per predictor (EPP). Often 10 EPP is considered sufficiently robust for predictor analysis. When calculating the power it seems we have a 64% power to detect a 20% increase or reduction in the hazard (HR<0.8 or HR>1.25). While this post hoc power calculation can be done it does not seem like good statistical practice (Hoenig & Heisey, 2001^[1]^). So instead in the manuscript we have now given reference to the sample size of similar studies that, in most cases, seem to include a sample of around 400 children treated. This is how it states now in the manuscript: “The study recruited a total of 801 children, which was an adequate sample size for analyzing predictors as judged by previous studies looking at time to recovery with samples sizes starting from 200 and most around 400 children treated [16–27].” (LINE 83-90 in final version without trackchanges)***

Reviewer #2: This study was an offshoot study from the Mango trial looking at the effect of a nutrient supplement on recovery from severe acute malnutrition

Firstly, the paper contains a number of grammatical errors that needs to be addressed.

***Response: Thank you for this note. We have now revised the manuscripts English thoroughly.***

Abstract/Introduction
More details need to be added on what SAM is, what is known about the causes of SAM etc.
What is not clear is if SAM can be caused by socioeconomic factors such as lack of food availability. This needs to be addressed as it ties into some of the speculations made in the discussion section in relation to categorizing the participants that present with illness vs those that don’t and their recovery.

***Response: Thank you for this comment. We have now included a paragraph on the causes of SAM in the Introduction section as follows: “Severe acute malnutrition (SAM) is a condition that occurs when the food intake does not meet the nutritional requirements either as a consequence of poor intake or disease [1]. Generally SAM arises in contexts with social, political and economic factors affecting food availability and where infections and inflammation are common [1]. This is also why no single intervention has been shown to reduce the incidence that requires a holistic package of interventions [2].” (LINE 43-47 in final version without trackchanges)***

4 million children where? Globally or locally?

***Response: Thank you for this comment. We now added the precision of “globally” in the following sentence: “Every year, more than 4 million children are treated for SAM globally*** ***[6] with a varying mean treatment time and proportion of non-response to treatment*** ***[7–11].” (LINE 57-58 in final version without trackchanges)***

Methods/results
It is unclear whether the data analysed are regarding standard RUTF or reduced RUTF

***Response: Thanks for the question. The data were analysed for all children treated regardless of their treatment arm. This has now been more clearly stated in the Methods section as follows: “All children included in the MANGO trial regardless of their treatment arm (reduced RUTF or standard RUTF) were included in the analysis.” (LINE 157-158 in final version without trackchanges)***

Also what is RUTF? A Nutrition label table should be added.

***Response: Thanks for the question. We have now added the following to the introduction section: “RUTF are energy and nutrient dense pastes usually composed of peanut butter, milk powder, oil, sugar and a vitamin and mineral complex designed to fulfil the nutritional needs of children recovering from SAM [4].” (LINE 51-53 in final version without trackchanges)***

Equations of WHZ and MUAC should be included

***Response: Thanks for the helpful comment. We have now added these precisions to the methods section under Data collection: “Weight was measured using an electronic scale (SECA 876) to the nearest 100 g, height using a wooden measuring board (locally made) to the nearest 1 mm, and MUAC using a non-stretchable colourless measuring tape to the nearest 1 mm. Z-scores were calculated using the WHO standards and STATA command zscore06 [21].” (LINE 115-118 in final version without trackchanges)***

Line 81: what does subsist mean?

***Response: Thanks for noting. We have changed the word to “depend” (LINE 100 in final version without trackchanges)***

Clearer detail on the study protocol should be included: Number of visits, when treatment was administered, dose, information collected, etc—in a figure.

***Response: Thanks for pointing out. We have added some clarifications for these points in the Methods section under the Study participants and treatment protocol and Data collection sub-sections. They detail when and what treatment was administered (specifically at admission and then during the weekly follow ups) and the type of data collected at different time points.***

Line 93: a reference needs to be included for the “national protocol”

***Response: Thank you. This has been done.***

Details of manufacturer for the kits/equipment used needs to be included in the “data collection” section

***Response: Thank you. These details have been included and the following additions/edits made to the sections: “Weight was measured using an electronic scale (SECA 876) to the nearest 100 g, height using a wooden measuring board (locally made) to the nearest 1 mm, and MUAC using a non-stretchable colourless measuring tape to the nearest 1 mm. Z-scores were calculated using the WHO standards and STATA command zscore06 [21].” (LINE 115-118 in final version without trackchanges)***

Line 108-109 how was data monitoring and cleaning performed?

***Response: Thank you for the comment. We have now added the following section to the Data collection sub-section of the Methods: “Data monitoring included among other thing, checking duplicate entries and outliers, anthropometric decimal distributions and correct prescription of medication according to diagnosed conditions. Any potential data problem resulted in action. Data cleaning was based on double checks of electronic data against patient registries or therapeutic cards.” (LINE 120-123 in final version without trackchanges)***

Not clear on how the predictor values were calculated in terms of hazards ratio, REF and odds ratio. Needs to be clarified.

***Response: Thank you for mentioning this shortcoming. We have now clarified this in the Data analysis sub-section of the Methods as follows: “Cox proportional hazard regression models were fitted to quantify effects of predictors on time to recovery by means of hazard ratios, describing the increased or decreased rate of change in likelihood of recovery.” (LINE 159-161 in final version without trackchanges)***

What is open defecation?

***Response: Thank you for the question. We have added the definition of open defecation in the Methods now as follow: “open defecation (the practice of defecating in nature instead of a toilet facility)” (LINE 150 in final version without trackchanges)***

1. John M. Hoenig; Dennis M. Heisey. The American Statistician, Vol. 55, No. 1. (Feb., 2001), pp. 19-24.
