# Supplementary material for: Predictors of time to recovery and non-response during outpatient treatment of severe acute malnutrition
Source: PLoS One. 2022 May 31;17(5):e0267538. doi: 10.1371/journal.pone.0267538 (PMC9154090; doi:10.1371/journal.pone.0267538)
Supplement: Attachment — Submitted filename: Response_to_Reviewers_5March2022.docx [file pone.0267538.s002.docx]

Dear Editor and Reviewers,

Many thanks for the careful review of our manuscript on the “Predictors of time to recovery and non-response during outpatient treatment of severe acute malnutrition”. We truly appreciated the thoughtful feedback provided and have now worked to improve our manuscript addressing the specific points raised. Please find below an explanation of the improvements made to the manuscript. Our responses are highlighted in ***bold italics***.

Editors comments:

Line 33. Suggest changing to  ..<-3, anaemia or no illness at admission…’  as it is now not clear whether the ‘no’ refers only to illness or also to anaemia.

***Response: Thanks for the observation. We have now changed the sentence as follows:***

***LINE 33: “…WHZ <-3, no illness nor anaemia at admission,..”***

Line 169-173. Can the authors explain the difference between ‘non-response to treatment’, which to me would indicate lack of weight gain, and ‘referred to inpatient care due to stagnant weight or weight loss’ (I leave out here the medical complications, which are different). Would the results have been different if children referred to inpatient care because of lack of weight gain would have been included in the analysis of time to recovery?

***Response: Thanks for the observation. Indeed we would expect that children ending up as non-responders would present with no weight gain during treatment. However, in our cohort we saw that some children continued to gain weight but with a very slow rate which meant that they were not classified as stagnant weights (where the definition was no more than 100g gained over 4 weeks) but continued their treatment. These children could also be gaining in height meaning that their WHZ was not improving which meant they were not reaching the recovery cut-off of WHZ-2. Also, to clarify the implication on the results of excluding the children referred to inpatient care we firsts added a sentence in the outcome definition section on line 142 and then a sentence on the sensitivity analysis results when including these children (which led to similar results).***

***Thus, we have now added the following sections:***

***LINE 101-103: “In case of medical complications, weight loss of over 5% at any point or stagnant weight defined as no more than 100g weight gained over 4 weeks, during treatment, children were referred to inpatient care, as per the Burkina national CMAM protocol.”***

***LINE 141-144: “For the study of time to recovery, non-recovered cases were right censored contributing to the analysis of time to recovery until exit from study. Patients referred to inpatient care were excluded from the analysis in order to limit potential bias that could be introduced due to short length of stay in treatment.”***

***LINE 210: “Including children referred to inpatient care in the analysis of different factors with time to recovery resulted in similar associations.”***

In table 4. How can sex and age still be in the model when the model was adjusted for these predictors (last columns)

***Response: Thanks for the question. The odds ratios for these variables (sex and age) in the sex and age adjusted model represent the OR adjusted for the other variable. Meaning, when looking at sex as a predictor, the adjusted model adjusts for age to look at the age independent predictive power of sex. And the inverse in the case of age. We have now made this clearer in all the tables (2, 3 and 4) adding a footnote indicating that “when analysing sex as a predictor, only age was included as adjustment and when analysing age as a predictor only sex was included as adjustment”.***

6. Review Comments to the Author

Reviewer #3: The study provides relevant information on a subject with limited literature. The publication of the study results could contribute to the current discussion on the need to further decentralise essential health services to reach a broader number of people in need.

Some aspects of this work could have been more clearly presented and explained, as detailed below:

Introduction

The authors should extend the rationale and explain why the topic treated is particularly crucial. This could be done by better explaining the absolute magnitude of the problem and, on the other hand, by better underling the implications of the study's results on current SAM treatment and prevention programs.

***Response: Thanks for the suggestion. We added the following sections highlighting the magnitude of the problem (in the 1^st^ paragraph of the introduction section) and the subsequent coverage of treatment (4^th^ paragraph of the introduction section):***

***LINE 43 – 49: “Severe acute malnutrition (SAM) is a condition that occurs when the food intake does not meet the nutritional requirements either as a consequence of poor intake or disease [1]. SAM is diagnosed when a child presents with a weight-for-height z-score (WHZ) <-3, a mid-upper-arm circumference (MUAC) <125 mm or nutritional oedema [2]. While the overall prevalence of SAM is unknown, in 2020 it was estimated that 2% of all children below the age of 5 years presented a WHZ <-3 translating to more than 13.6 million children suffering from severe wasting at any time [3]. Children with SAM have a 11.6 increased risk of mortality compared to children with no nutritional deficits living in the same contexts [4].”***

***LINE 62-66: “In 2020, around 5 million children were treated for SAM globally [10]. With 13.6 million children suffering from severe wasting at any time and applying of incidence correction factor of 3.5 [11] to account for all new cases arising in a year, this translates to 47.6 million episodes of severe wasting in a year. Thus, the current coverage of treatment is around 10%. This is when excluding the burden of MUAC cases which, if added, would translate to an even lower coverage of treatment.*** ***Such low coverage warrants for reflection on how to improve it possibly by optimising and better targeting treatment in order to expand it to more cases.”***

In the background, the authors mentioned the last figure available for coverage without mentioning the global burden of wasted children 6-59months.

***Response: Thanks for the observation. We added the following sections highlighting the magnitude of the problem (in the 1^st^ paragraph of the introduction section) and the subsequent coverage of treatment (4^th^ paragraph of the introduction section):***

***LINE 43 – 49: “Severe acute malnutrition (SAM) is a condition that occurs when the food intake does not meet the nutritional requirements either as a consequence of poor intake or disease [1]. SAM is diagnosed when a child presents with a weight-for-height z-score (WHZ) <-3, a mid-upper-arm circumference (MUAC) <125 mm or nutritional oedema [2]. While the overall prevalence of SAM is unknown, in 2020 it was estimated that 2% of all children below the age of 5 years presented a WHZ <-3 translating to more than 13.6 million children suffering from severe wasting at any time [3]. Children with SAM have a 11.6 increased risk of mortality compared to children with no nutritional deficits living in the same contexts [4].”***

***LINE 62-66: “In 2020, around 5 million children were treated for SAM globally [10]. With 13.6 million children suffering from severe wasting at any time and applying of incidence correction factor of 3.5 [11] to account for all new cases arising in a year, this translates to 47.6 million episodes of severe wasting in a year. Thus, the current coverage of treatment is around 10%. This is when excluding the burden of MUAC cases which, if added, would translate to an even lower coverage of treatment.*** ***Such low coverage warrants for reflection on how to improve it possibly by optimising and better targeting treatment in order to expand it to more cases.”***

The comparison between global burden and treatment coverage shows how coverage is still impressively low. Thus, how it is essential to reach more children affected by SAM. As mentioned in the paper, a shorter treatment length would eventually decrease costs per individual treated. Reduced cost per child treated could subsequently help extend the coverage of the programs.

***Response: Indeed! To make this clear in the manuscript, we added the following paragraph in the Introduction section:***

***LINE 62-66: “In 2020, around 5 million children were treated for SAM globally [10]. With 13.6 million children suffering from severe wasting at any time and applying of incidence correction factor of 3.5 [11] to account for all new cases arising in a year, this translates to 47.6 million episodes of severe wasting in a year. Thus, the current coverage of treatment is around 10%. This is when excluding the burden of MUAC cases which, if added, would translate to an even lower coverage of treatment.*** ***Such low coverage warrants for reflection on how to improve it possibly by optimising and better targeting treatment in order to expand it to more cases.”***

The authors should also consider explaining that global data reported are only for children 6-59 months and eventually why this target group is so critical.

***Response: Thanks for the comment. We added the following sentence to the 1^st^ paragraph of the introduction section to clarify the importance of targeting children with SAM due to their high risk of mortality:***

***LINE 49: “Children with SAM have a 11.6 increased risk of mortality compared to children with no nutritional deficits living in the same contexts [4].”***

Methods
Statistical analysis is clearly described.

***Response: Thanks!***

Discussion
Findings are clearly resumed and discussed.

***Response: Thanks!***

Minor points:
-Consider eventually modifying the abstract according to the modifications done in the introduction

***Response: Due to very limited space in abstract, we did not add any of the further justifications now included in the introduction section.***

- line 150, it would be better to specify what acronym ARI is the first time it is used. The authors specified it only a few lines below.

***Response: Thanks for the observation. We defined ARI now at the 1^st^ appearance on LINE 150.***
